# Current News

**Published:** 1892-02

**Authors:** 


### Current News.

### ODONTOGRAPHIC SOCIETY OF CHICAGO.

AT the annual meeting of the Odontographic Society of Chicago, held December 14, 1891, the following officers were elected for the ensuing year:

President, E. L. Clifford; Vice-President, Geo. J. Dennis; Recording Secretary, U. G. Poyer; Corresponding Secretary, T. A. Broadbent; Treasurer, E. Noyes.

*Board of Directors.*—E. L. Clifford, Geo. J. Dennis, U. G. Poyer, R. B. Tuller, C. E. Bentley.

*Board of Censors.*—D. C. Bacon, Louis Ottofy, D. M. Gallie.

T. A. Broadbent,
*Corresponding Secretary.*

---

### ANNUAL MEETING OF THE LOUISIANA STATE DENTAL SOCIETY.

The Annual Meeting of the Louisiana State Dental Society will take place at New Orleans on March 2, 1892.

The profession at large is invited to attend. The date of the meeting is the day following Mardi Gras, and at which time very liberal rates can be secured.

Dr. J. E. Dugger,
*Corresponding Secretary.*

